# Giant photovoltaic response in band engineered ferroelectric perovskite

**DOI:** 10.1038/s41598-018-26205-x

**Published:** 2018-05-22

**Authors:** Subhajit Pal, Atal Bihari Swain, Pranab Parimal Biswas, D. Murali, Arnab Pal, B. Ranjit K. Nanda, Pattukkannu Murugavel

**Affiliations:** Department of Physics, Indian Institute of Technology Madras, Chennai, 600036 India

## Abstract

Recently the solar energy, an inevitable part of green energy source, has become a mandatory topics in frontier research areas. In this respect, non-centrosymmetric ferroelectric perovskites with open circuit voltage (*V*_OC_) higher than the bandgap, gain tremendous importance as next generation photovoltaic materials. Here a non-toxic co-doped Ba_1−*x*_(Bi_0.5_Li_0.5_)_*x*_TiO_3_ ferroelectric system is designed where the dopants influence the band topology in order to enhance the photovoltaic effect. In particular, at the optimal doping concentration (*x*_*opt*_ ~ 0.125) the sample reveals a remarkably high photogenerated field *E*_OC_ = 320 V/cm (*V*_OC_ = 16 V), highest ever reported in any bulk polycrystalline non-centrosymmetric systems. The band structure, examined through DFT calculations, suggests that the shift current mechanism is key to explain the large enhancement in photovoltaic effect in this family.

## Introduction

The advancement of semiconductor *p*-*n* junction solar energy in last few decades played a pivotal role as a clean energy resource. However, the open circuit voltage (*V*_OC_) being lower than the band gap (*E*_g_), in these materials, posed obstacle for the future development of *p-n* junction solar cell^1^. Several new materials have been studied for their improved efficiency and photovoltaic (PV) characteristics^2–5^. Interestingly, ferroelectric oxides are reported to show anomalous PV effect with above bandgap voltage^6–10^. In fact, Glass *et al*. observed giant photovoltaic response in Fe doped LiNbO_3_ single crystal with photogenerated field of the order of 10^4^ V/cm^9^. The origin of such anomalous photovoltaic effect is generally ascribed to ballistic and shift current phenomena^11–14^. However, the progress of this field demands research on wide range of ferroelectric materials. So far, the PV studies are limited to only few ferroelectrics, namely (Pb,La)(Zr,Ti)O_3_^6^, BaTiO_3_ (BTO)^8^, Fe:LiNbO_3_^9^, PbTiO_3_^12^, (K,Ba)(Nb,Ni)O_3_^15^, BiFeO_3_^16^, Pb(Zr,Ti)O_3_^17^, KBiFe_2_O_5_^18^. The observed PV in these ferroelectrics is an outcome of inter-band carrier transitions and hence is largely dependent on the extent of delocalization of valence and conduction states which in turn is determined through the strength of covalent bonding^19^. Among ferroelectric ABX_3_ perovskites, the tetragonal BTO shows poor PV response due to localized Ti *d* orbital forming the conduction band edge (CBE) states^12^, whereas CH_3_NH_3_PbI_3_ shows better PV response due to delocalized and spin orbit coupled *p* states of Pb^2+^ ion in CBE^20^. In fact previous reports show that the shift current response is highly dependent on the orbital character of CBE^20,21^. If the orbitals forming the CBE has same orientation as that of the polarization direction, there is large shift current which in turn enhances the PV response. For example studies on rhombohedral (K,Ba) (Nb,Ni)O_3*−δ*_, where the polarization direction is along the body diagonal, show the degenerate *t*_2g_ orbitals (*xy*, *xz* and *yz*) at CBE which results in large shift current response. Whereas, for the tetragonal structure with polarization along *z*, the *xy* orbital forms the CBE leading to a low shift current response^21^.

In this work, we have engineered the band structure of the well-known non-centrosymmetric BTO through doping so that the delocalized CBE is formed by the orbitals oriented along the direction of polarization. Both Bi and Pb are found to be favourable dopants at the A-site and form covalent bonding with the O-*p* states so that the states in the vicinity of the Fermi level can be made more delocalized^22,23^. Pb being toxic Bi is preferred as dopant. With Bi^3+^ doping at Ba^2+^ site, a monovalent co-dopant is necessary for charge compensation. In this regards we find Li is an appropriate co-dopant as it is completely ionic and does not affect the frontier electronic structure. We demonstrate that the Bi and Li co-doped BTO samples reveal enhancement in *V*_OC_ with composition, the highest value being 16 V for the optimal composition *x* = 0.125.

## Results and Discussion

**Figure 1:**
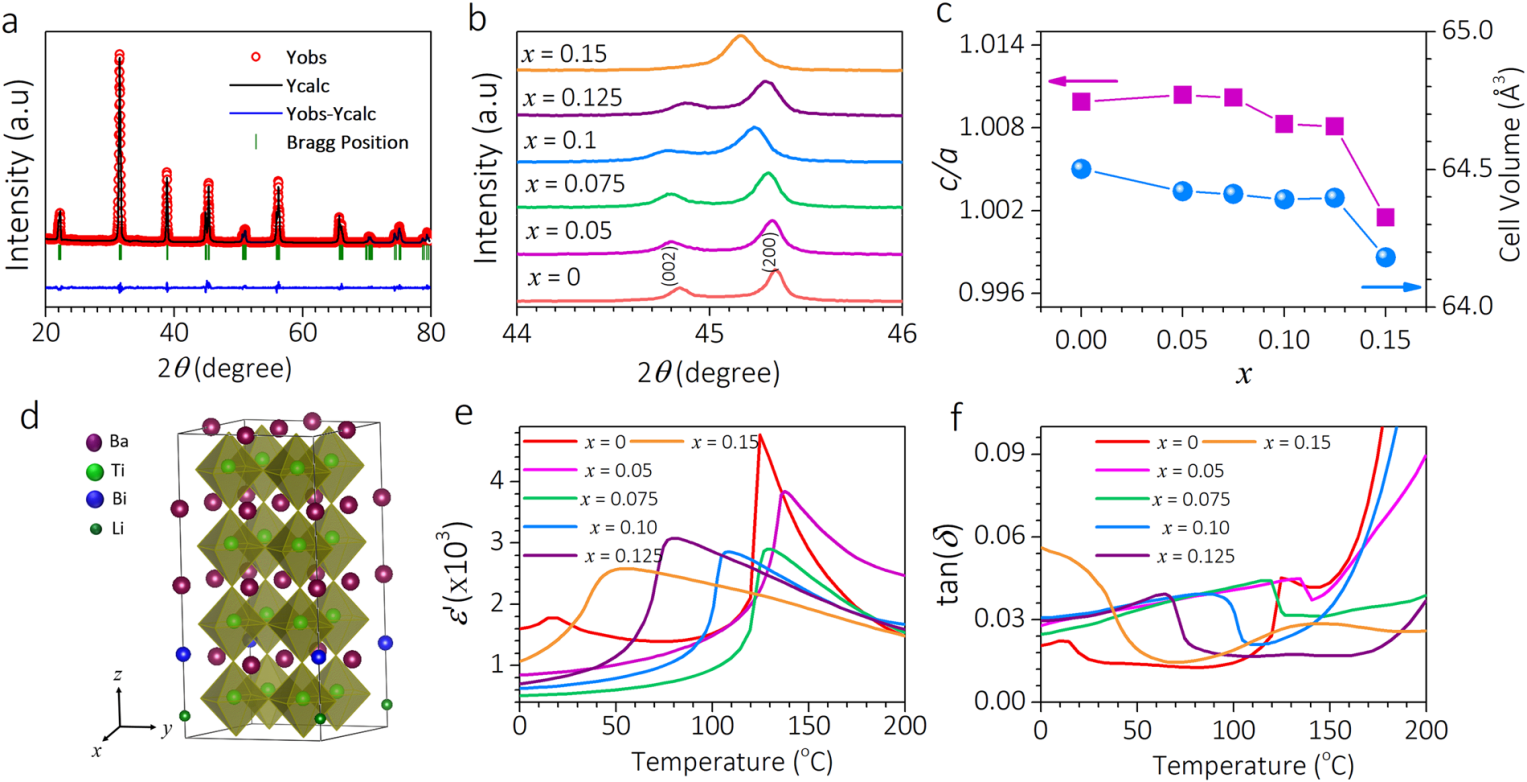
Structural and dielectric properties of BBLT. (**a**) X-ray diffraction and Rietveld refined pattern of BBLT for *x* = 0.125. (**b**) The evolution of (002) and (200) peaks with composition *x*. The gradual merging of these two peaks with higher concentration suggest the reduction of tetragonality. (**c**) The *c*/*a* and cell-volume plotted as a function of *x*. (**d**) Relaxed 224 supercell structure representing *x* = 0.125. (**e**) The *ε*′ versus temperature curves at 1 kHz showing the ferroelectric transition. (**f**) Dielectric loss as a function of temperature.

The Ba_1−*x*_(Bi_0.5_Li_0.5_)_*x*_TiO_3_ (BBLT) samples, *x* = 0.0, 0.05, 0.075, 0.1, 0.125 and 0.15, are synthesized by solid-state method. X-ray diffraction (XRD) patterns for *x* = 0.125 (Fig. 1(a)), 0.0, 0.05, 0.075, 0.1, and 0.15 (Supplementary Information Fig. 1(a) to (e)) show pure tetragonal phase with *P4mm* space group as confirmed by Rietveld refinements. For large doping concentration (*x* > 0.125) the tetragonality is reduced which is illustrated through merging of peaks (Fig. 1(b)) and is further reaffirmed by the ratio of lattice parameters, *c*/*a*, approaching towards unity (Fig. 1(c)). However, the *c*/*a* ratio shows a subtle increment for *x* = 0.05 and thereafter decreasing trend with *x* (Fig. 1(c)) which can be correlated with tilting of oxygen octahedra associated with expansion of the lattice constant *a* in the region of 0.075 ≤ *x* ≤ 0.15^24^. The decrease in cell-volume with *x* (Fig. 1(c)) is due to the smaller ionic radii of the dopants. The temperature variation of real part of permittivity (*ε*′) and loss factor (tan*δ*) at 1 kHz from 0 to 200 °C (Fig. 1(e),(f)) unveil the ferroelectric to paraelectric transition (*T*_C_) which is at 124 °C for BTO; increased to 137 °C for *x* = 0.05 and thereafter decreased to 129, 108, 79, and 55 °C for *x* = 0.075, 0.10, 0.125, and 0.15, respectively. The change in *T*_C_ with composition is in accordance with change in *c*/*a* ratio (Fig. 1(c)) as reported in several A-site doped BTO samples^24–26^. Additionally, we find that *x* = 0.15 composition exhibits relaxor behaviour as evidenced from the shift in *T*_C_ with frequency (Supplementary Information Fig. 2).

**Figure 2:**
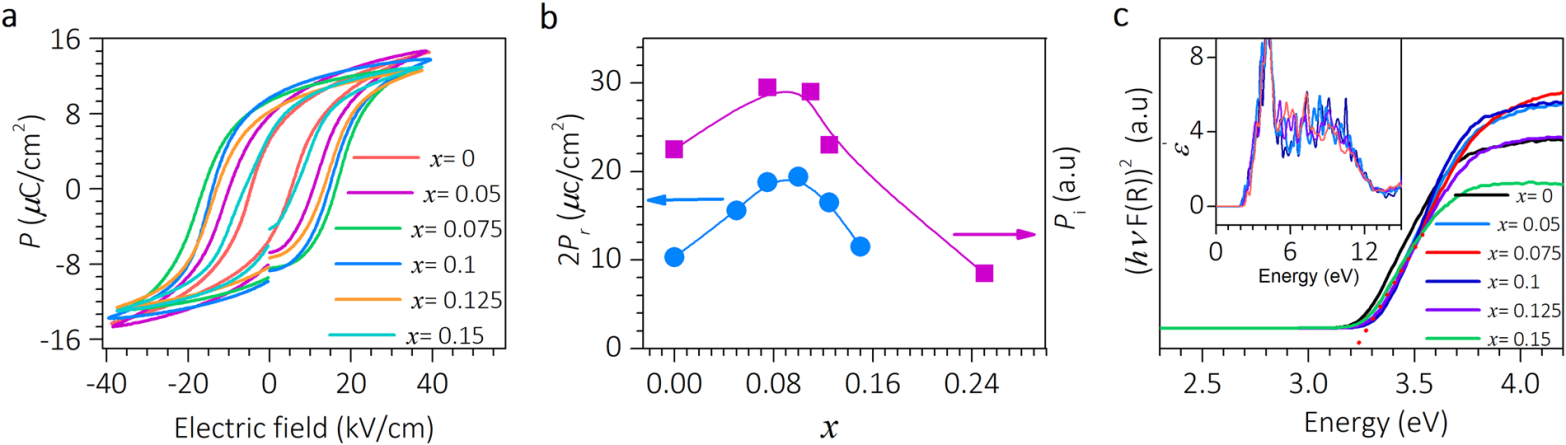
Ferroelectric and optical properties of BBLT. (**a**) *P*-*E* hysteresis loops for samples at room temperature. (**b**) DFT calculated ionic dipole moment and compositional variations of 2*P*_*r*_. (**c**) Diffuse reflectance spectra of BBLT samples and inset shows DFT derived optical absorption spectra for bulk and doped samples.

The polarization versus electric field hysteresis loops measured at 4 Hz (Fig. 2(a)) reiterates the prevailing ferroelectric state of all samples. The remnant polarization (2*P*_r_) is enhanced compared to BTO value for each doping concentration (Fig. 2(b)). Notably, *x* = 0.10 composition exhibits 2*P*_r_ = 19.5 *μ*C/cm^2^ which is nearly 100% more than BTO. In fact, our first principle calculations demonstrate that the total ionic dipole moments *p*_i_ follow similar trend with composition (Fig. 2(b)). The samples show nearly invariant optical bandgap of around 3.2 eV, as deduced from Kubelka-Munk plot based on diffused reflectance spectrum (Fig. 2(c))^27,28^. The optical absorption spectra measured from DFT calculations further confirms the invariance (Fig. 2(c), inset).

**Figure 3:**
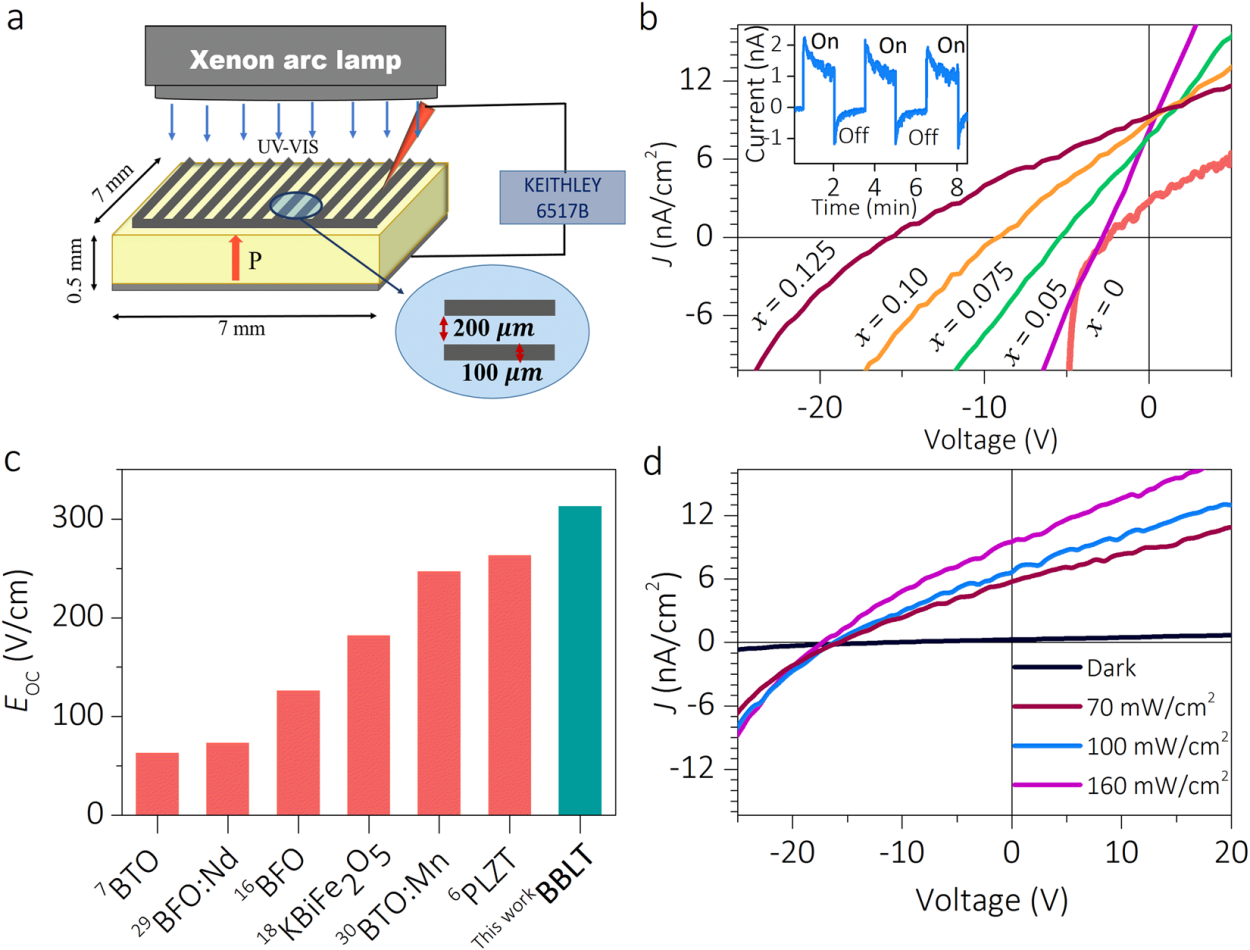
Photovoltaic measurements on the BBLT sample. (**a**) The experimental geometry used for PV measurements. (**b**) The *J-V* characteristic plots displaying the PV response of all compositions. The inset shows photocurrent response with time for *x* = 0.125. (**c**) The histogram comparing *V*_OC_ (normalized with respect to thickness) of BBLT with that of well-known ferroelectrics reported in the literature. (**d**) The *J-V* measured under different illumination intensity for *x* = 0.125.

To examine the photovoltaic response of the BBLT samples, the adopted capacitor includes a finger geometry as top electrode (Fig. 3(a)) and the current density (*J*) versus bias voltage (*V*) is measured under dark and 160 mW/cm^2^ light illumination (Fig. 3(b)). Upon co-doping, there is a remarkable enhancement in *V*_OC_ (Supplementary Information Fig. 3). In fact, *x* = 0.125 composition (Fig. 3(b)) exhibits the maximum *V*_OC_ of 16 V (*E*_OC_ = 320 V/cm) with *J*_SC_ = 9.18 nA/cm^2^. To the best of our knowledge the displayed *V*_OC_ of the present polycrystalline sample (*x* = 0.125) brings 2 fold increase in the earlier reported value on single crystalline BTO^8^. According to the comparison histogram (Fig. 3(c)) it is indeed the largest ever reported *V*_OC_ for any bulk polycrystalline ferroelectric oxides including few single crystalline compounds^6,7,16,18,29,30^. Surprisingly, even though *x* = 0.15 composition has polarization the PV response almost ceases to exist (Supplementary Information Fig. 4(d)). It suggests that there is a simultaneous effect of lattice polarization and conduction band topology to create an optimum composition (*x* ~ 0.125) for which the PV response is maximum as will be understood from the DFT calculations.

**Figure 4:**
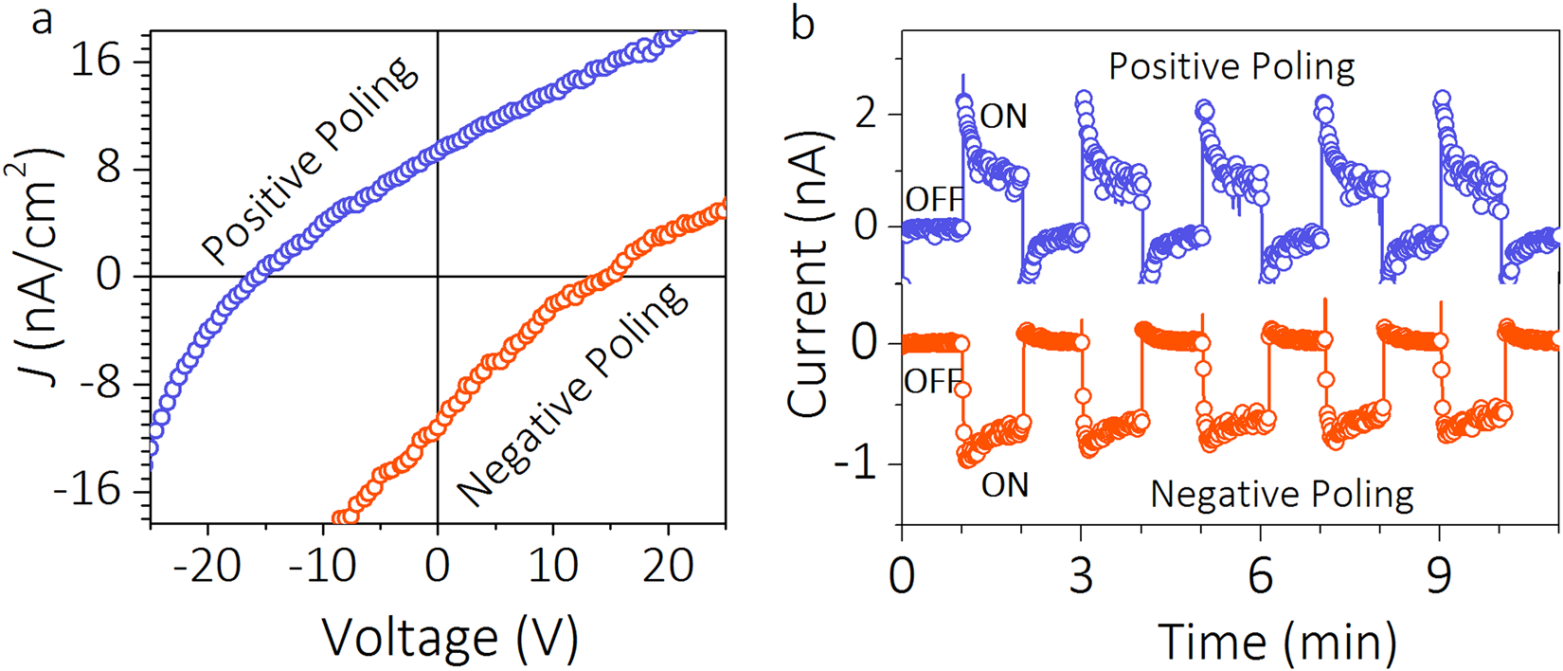
Switchable photovoltaic effect of *x* = 0.125 sample. (**a**) *J-V* characteristic under upward and downward poling condition. (**b**) Time dependent photocurrent response under different poling directions.

The photocurrent response under zero bias measured in light ON and OFF states for *x* = 0.125 (Fig. 3b), 0.05, 0.075, 0.10 and 0.15 (Supplementary Information Fig. 4(a) to (d)) samples confirm a quick photocurrent response. The spikes observed are due to the pyroelectric response^6^. Notably, the PV response of *x* = 0.125 sample illustrates a systematic increase in *J*_SC_ with light intensity while *V*_OC_ remains constant (Fig. 3(d)). Although the light polarization dependent PV response could provide direct evidence for bulk photovoltaic effect (BPVE), the *V*_OC_ being independent of light intensity can also indicate the signature of BPVE^8,11,31,32^. To investigate the photovoltaic response on polarization state, *J-V* measurements and time dependent photocurrent responses are carried out on samples subjected to positive and negative poling fields. The respective plots for *x* = 0.125 are shown in Fig. 4(a),(b). The plots reveal a sign change on *V*_OC_ and *J*_SC_ with change in poling state. The observed switchable PV response elucidates the major role of polarization than the electrode contribution.

Since BPVE is largely influenced by the band structure in the vicinity of the Fermi level, we have carried out band structure calculations using DFT (see computational method) to examine the role of the dopants in enhancing the PV response. Bader charge of the ions (Table 1) and deviation of this from the ideal charge state provides a measure of covalency. As the table shows, Ti^4+^ and O^2−^ exhibit a significant deviation of ~1.9 e and 0.8 e respectively, to suggest that there are reasonable *p*-*d* and *p*-*p* covalent interactions^12^. On the other hand for Ba^2+^, the deviation is small (0.44 e) which suggest that Ba is more ionic. Now with Bi doping, the covalent interaction at the A-site increases significantly as it brings deviation as large as ~1.2 e.

**Table 1:** Ionic charges for BBLT as estimated from the Bader charge analysis.

*x*	Li^1+^	Bi^3+^	Ba^2+^	Ti^4+^	O^2−^
0	—	—	1.604	2.103	−1.20
0.075	0.913	1.821	1.558	2.128	−1.227
0.125	0.914	1.818	1.563	2.103	−1.214
0.25	0.922	1.817	1.556	2.123	−1.213

The conventional charge state of the ions are mentioned in superscript. The deviation from the conventional charge state is a measure of covalency.

**Figure 5:**
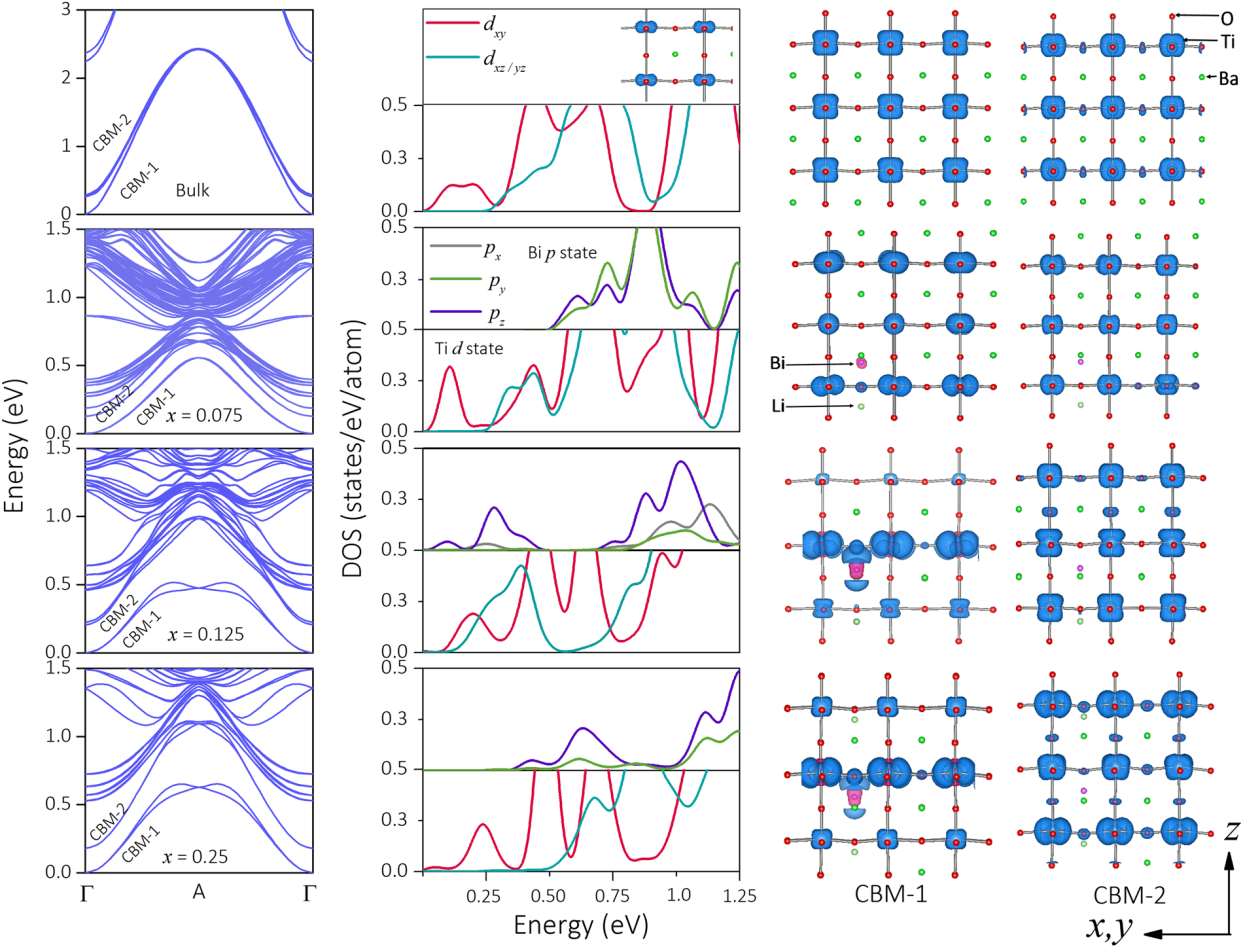
Electronic structure of BBLT. First column: Spin-orbit coupled conduction band structure of BBLT as a function of *x*. The conduction band minimum (CBM) is set to zero. CBM-1 and CBM-2 are self-explanatory. Second column: The corresponding orbital resolved Ti-*t*_2g_ and Bi-*p* DOS in the conduction band spectrum. The charge densities (iso-value = 6 × 10^−3^ eV/Å^3^) for CBM-1 and CBM-2 are shown in third and fourth columns respectively. The orbital characters of CBM-1 and CBM-2 changes with *x*. While in the bulk Ti*-xy* state dominates CBM-1 (see inset), the *z*-axis oriented orbitals (*xz* and *yz*) become more prominent in CBM-1 with increase in *x*. However, for larger doping (*x* = 0.25) the CBM-1 is again dominated by the planar *xy* orbital. The dopant Bi-*p* characters increases their contribution to CBM-1 with increase in *x*.

The covalent interactions are reflected in the band dispersion. In bulk BTO, the lower lying conduction bands (CBM-1 and CBM-2) (Fig. 5, first row) are occupied by the antibonding Ti-*t*_2g_ states^33^. The polarized BTO, breaks the cubic symmetry to lower the Ti*-xy* state which now dominates the CBM-1, while *xz* and *yz* states occupy CBM-2. However, with doping of Bi and Li, the nature of Ti-O polarization around the dopant neighborhood changes as the TiO_6_ complex is distorted. This leads to a significant presence of *xz* and *yz* orbitals in CBM-1. For *x* = 0.125 (third row, Fig. 5), the CBM-1 and CBM-2 are occupied by all the *t*_2g_ states. However, for *x* = 0.25 (fourth row, Fig. 5), we find that the CBM is again dominated by the *xy* states while CBM-2 is by the other *t*_2g_ characters. Beside Ti-O polarization, the doping brings an additional Bi-O polarization along the *z*-axis and it breaks the three fold degeneracy of Bi-*p* states. As seen from the Bi-*p* DOS and charge densities (Fig. 5), the contribution of the *p*_z_ state in CBM-1 gradually increases with doping. Additionally, the shape of the charge densities highlights the Bi-O covalent interaction. A larger doping concentration strengthens both Bi-O polarization and Bi-O interaction. As a consequence the antibonding Bi-*p* states lie above the CBE and do not contribute to PV response. Also we find that large Bi-O polarization decreases the Ti-O bond length along *z* leaving the *xy* states lower in energy to form the CBE which further decreases the PV response supporting the experimental results.

According to shift current theory, if the CBE is more occupied by *z*-axis oriented orbitals (e.g. *p*_z_, *xz*, *yz*, *z*^2^*−1*) and is very delocalized, resulting from stronger covalent interaction along the polarization direction, the shift current response is high and a large BPVE is observed^12^. As discussed above, in the case of BBLT, the following trends are observed. (I) Initially the polarization increases with *x* and peaks around *x* = *x*_*opt*_ (~0.125) and decreases afterwards. (II) The covalency increases with *x* and it is attributed to Bi-*p* – O-*p* interactions. (III) The conduction band edge is more occupied by the *z*-axis oriented orbitals (Bi-*p*_*z*_, Ti- *xz* and *yz*) and the contribution become maximum around *x* = *x*_*opt*_. Therefore, based on shift current theory, the BPVE should increase with *x* and become maximum at *x*_*opt*_. We may note that since the calculations are carried out for some discrete values of *x*, exact value of *x*_*opt*_ cannot be determined theoretically. However, experimentally we find that the BPVE is maximum at *x* = 0.125.

## Conclusion

In summary, we have demonstrated a unique way to tailor the bands via A-site doping to create giant photovoltaic response in polycrystalline perovskite ferroelectric oxides. In the present work, this has been achieved by co-doping Bi and Li in BaTiO_3_. This work opens up an opportunity to design new family of ferroelectric compounds for photovoltaic studies by carefully choosing the dopants in such a way that it will lead to simultaneous increase in lattice polarization and delocalization of the conduction band edge state.

## Materials and Methods

### Experimental section

Ba_1−*x*_(Bi_0.5_Li_0.5_)_*x*_TiO_3_ with composition *x* = 0.0, 0.05, 0.075, 0.1, 0.125 and 0.15 were fabricated by solid state method. Stoichiometric mixture of initial precursors BaCO_3_ (purity ≥ 99.9%), TiO_2_ (purity ≥ 99.9%), Bi_2_O_3_ (purity ≥ 99.9%) and Li_2_CO_3_ (purity ≥ 99.9%) were ground in an agate mortar and calcined at 700 °C for 4 h followed by 800 °C for 6 h. The uniaxially pressed pellets of 12 mm diameter made from calcined powder of various composition were fired at optimized temperatures in the range of 1000 to 1300 °C for 2 h. The powdered samples were subjected to X-ray diffraction (Rigaku smartLab) experiments for phase confirmation. The Rietveld refinement was done on the XRD pattern by FULLPROF software. For dielectric and polarization measurements, the 12 mm diameter sintered pellets were coated with Ag as electrodes. The temperature dependent dielectric permittivity and loss factor were obtained from Novocontrol impedance analyser in 10 Hz to 10 MHz range. The polarization measurements were carried out by employing Radiant Technology loop tracer at 27 °C. The Ultraviolet–Visible–Near Infrared (UV–VIS–NIR) Spectro-Photometer (JASCO V-650) was employed for optical band gap measurements. For PV measurements, the polished samples were cut into 7 × 7 mm square and 0.5 mm thickness dimension. On the top side of the sample, silver electrodes of size 6.4 × 6.4 mm (finger thickness 100 *μ*m and gap 200 *μ*m) were deposited by thermal evaporation technique using shadow mask and the bottom side is covered by Ag paint. The samples were poled by 20 kV/cm field in pressure contact mode over entire electrode area using a thin copper plate of 0.15 mm thickness on the top finger electrode. The effective poling was confirmed by comparing the *d*_33_ values with and without finger electrode. Upward/downward poling implies the application of a negative/positive voltage to the top electrode. PV measurements were carried out by employing Xenon-arc lamp (Newport, Model No-67005) as light source and Keithley electrometer (6517B) as measuring unit. The values of current density, *J*_SC_, were arrived by considering the entire top surface area (7 × 7 mm^2^) in the calculation.

### Computational method

Present DFT calculations are performed using Pseudopotential (PP) based Vienna *ab-initio* Simulation Package (VASP)^34^. PPs are based on projected augmented wave (PAW) method with exchange and correlation effects described using Generalised Gradient Approximation (GGA) and Perdew-Burke-Ernzerhof (PBE) functional^35,36^. For the elements present in our model, the following valance electrons were explicitly considered in the PP: Ba 4 *s*^2^ 4*p*^6^ 5 *s*^2^, Li 1 *s*^2^ 2s^1^, Bi 5*d*^10^ 6 *s*^2^ 6*p*^3^, Ti 3*p*^6^ 3*d*^2^ 4 *s*^2^ and O 2 *s*^2^ 2*p*^4^. In order to account for relativistic effects arising from heavy Bi element, spin orbit coupling (SOC) has been included in our calculations. SOC calculations were performed as implemented in PAW methodology in VASP package^37,38^. Since SOC is predominantly act in the immediate vicinity of nuclei, the SOC Hamiltonian λL.S is solved self consistently along with PAW Hamiltonian within the PAW sphere. The plane wave cut-off energy was chosen as 500 eV. To simulate experimental doping concentrations we adopted supercell approach with periodic boundary conditions and performed our calculations using 3 × 3 × 3, 3 × 3 × 2, 2 × 2 × 4 and 2 × 2 × 2 supercells corresponding to *x* = 0.07, 0.11, 0.125, and 0.25 respectively. In each of these supercells, two Ba ions are replaced by single Bi and Li ions. We performed our calculations with Li and Bi in four different orientations [001], [100], [110], [111] corresponding to Bi and Li bond lengths 4.03 Å, 3.98 Å, 5.63 Å, 6.94 Å respectively. The total energy and band structure of these configurations are found to be nearly identical. Therefore, in the present work only one of them [001] is presented in detail. For structural optimization, we chose a convergence criterion of 10^−6^ eV for self-consistent field (SCF) electronic energy and 10^−3^ eV/Å for Hellmann–Feynman forces on each atom. A 8 × 8 × 8 Monkhorst–Pack grid is used for the Brillouin Zone integration of bulk BaTiO_3_^39^. Proportionate *k*-grids are used for the supercells. The total ionic charges were calculated using the Bader Atom Molecule (AIM) approach as implemented in the program by Arnaldsson *et al*.^39,40^.

## Electronic supplementary material

Supplementary materials
