# Supplementary material for: Giant photovoltaic response in band engineered ferroelectric perovskite
Source: Sci Rep. 2018 May 22;8:8005. doi: 10.1038/s41598-018-26205-x (PMC5964148; doi:10.1038/s41598-018-26205-x)
Supplement: Supplementary file 1 — Supplementary materials [file 41598_2018_26205_MOESM1_ESM.docx]

**Supplementary materials for**

**“Giant photovoltaic response in band engineered ferroelectric perovskite”**

Subhajit Pal, Atal Bihari Swain, Pranab Parimal Biswas, D. Murali, Arnab Pal, B. Ranjit K. Nanda*, and Pattukkannu Murugavel**

**

**Department of Physics, Indian Institute of Technology Madras, Chennai 600036, India

**Supplementary Figure 1. Rietveld refinement of Ba_1-_*_x_*(Bi_0.5_Li_0.5_)*_x_*TiO_3_** (**BBLT) obtained from FULLPROFF software.** The experimental XRD data (red circle), refinement data (black line) and their differences (blue line) along with the Bragg positions (green mark) are plotted for the compositions **(a)** *x* = 0.0, **(b)** 0.05, **(c)** 0.075, **(d)** 0.10, and **(e)** 0.15.

**Supplementary Table 1. Structural and fitted parameter of BBLT samples.** The lattice parameters (*a* and *c*), unit cell volume (*v*), weighted profile *R* factor (*R*_wp_), expected *R* factor (*R*_exp_) and goodness of fit (*S* = *R*_wp_/*R*_exp_) extracted from Rietveld refined XRD patterns for BBLT compositions. The satisfactory *S* values suggest a good quality of the structural refinement.

| Parameters | *x* = 0.0 | *x* = 0.05 | *x* = 0.075 | *x* = 0.1 | *x* = 0.125 | *x* = 0.15 |
| --- | --- | --- | --- | --- | --- | --- |
| *a* ([Å](https://en.wikipedia.org/wiki/%C3%85)) | 3.9972 | 3.9949 | 3.9949 | 3.9971 | 3.9975 | 4.0018 |
| *c* ([Å](https://en.wikipedia.org/wiki/%C3%85)) | 4.0370 | 4.0370 | 4.0360 | 4.0304 | 4.0298 | 4.0083 |
| *v* ([Å](https://en.wikipedia.org/wiki/%C3%85)^3^) | 64.501 | 64.42 | 64.41 | 64.39 | 64.39 | 64.18 |
| *R*_wp_ (%) | 9.89 | 10.1 | 9.71 | 10.4 | 10.4 | 11.3 |
| *R*_exp_ (%) | 5.31 | 5.48 | 5.98 | 5.23 | 5.47 | 5.64 |
| *S* | 1.86 | 1.84 | 1.62 | 1.98 | 1.90 | 2.00 |

**

**

**Supplementary Figure 2. Dielectric plot of BBLT for *x* = 0.15 composition.** The temperature variation of real part of permittivity (*ε*′) shows the shift in *T*_C_ towards high temperature with increase in frequency which indicates the relaxor characteristic.



**

Supplementary Figure 3. The observed *V*_OC_ for the BBLT sample for all compositions.** The *V*_OC_ plotted for all compositions reveals increasing trend with maximum value of 16 V observed for *x* = 0.125 composition.

**Supplementary Figure 4. Photocurrent response of BBLT samples.** The photocurrent response under zero bias during light ON and OFF state plotted with respect to time for compositions **(a)** *x* = 0.05 **(b)** *x* = 0.075 **(c)** *x* = 0.1 and **(d)** *x* = 0.15. As explained in the main text, the composition *x* = 0.15 does not show any photoresponse.

**
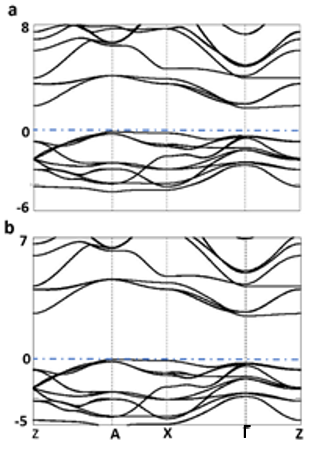
**

**Supplementary Figure 5. Band structure of BTO along high symmetry direction. (a)** DFT + SOC band structure with band gap ~ 1.9 eV and **(b)** DFT + SOC + HSE band structure with band gap of ~ 3.2 eV. GGA is known to underestimate the Bandgap which can be further improved through Heyd–Scuseria–Ernzerhof (HSE06) hybrid functional. For bulk BTO, the calculated bandgap of ~ 3.2 eV using HSE06 functional show good agreement with experiment. However, HSE functional have known problem of incorrectly predicting defect states^32^. Hence for doped system, present calculation were performed using state of the art GGA – PBE functionals.

41. Nieminen, R. M. Issues in first-principles calculations for defects in semiconductors and

Oxides. Modelling Simul. Mater. Sci. Eng. **17,** 084001 (2009).
